# Antioxidant Properties of Second-Generation Antipsychotics: Focus on Microglia

**DOI:** 10.3390/ph13120457

**Published:** 2020-12-12

**Authors:** Giuseppe Caruso, Margherita Grasso, Annamaria Fidilio, Fabio Tascedda, Filippo Drago, Filippo Caraci

**Affiliations:** 1 Department of Drug Sciences, University of Catania, 95125 Catania, Italy; grasso.margherita@studium.unict.it (M.G.); fcaraci@unict.it (F.C.); 2 Department of Laboratories, Oasi Research Institute—IRCCS, 94018 Troina, Italy; 3 Department of Biomedical and Biotechnological Sciences, University of Catania, 95123 Catania, Italy; anna.fidilio@unict.it (A.F.); fdrago@unict.it (F.D.); 4 Department of Life Sciences, University of Modena and Reggio Emilia, 41125 Modena, Italy; tascedda@unimore.it; 5 Center for Neuroscience and Neurotechnology, University of Modena and Reggio Emilia, 41125 Modena, Italy

**Keywords:** oxidative stress, schizophrenia, dopamine, antioxidants, antipsychotics, inflammation

## Abstract

Recent studies suggest a primary role of oxidative stress in an early phase of the pathogenesis of schizophrenia and a strong neurobiological link has been found between dopaminergic system dysfunction, microglia overactivation, and oxidative stress. Different risk factors for schizophrenia increase oxidative stress phenomena raising the risk of developing psychosis. Oxidative stress induced by first-generation antipsychotics such as haloperidol significantly contributes to the development of extrapyramidal side effects. Haloperidol also exerts neurotoxic effects by decreasing antioxidant enzyme levels then worsening pro-oxidant events. Opposite to haloperidol, second-generation antipsychotics (or atypical antipsychotics) such as risperidone, clozapine, and olanzapine exert a strong antioxidant activity in experimental models of schizophrenia by rescuing the antioxidant system, with an increase in superoxide dismutase and glutathione (GSH) serum levels. Second-generation antipsychotics also improve the antioxidant status and reduce lipid peroxidation in schizophrenic patients. Interestingly, second-generation antipsychotics, such as risperidone, paliperidone, and in particular clozapine, reduce oxidative stress induced by microglia overactivation, decreasing the production of microglia-derived free radicals, finally protecting neurons against microglia-induced oxidative stress. Further, long-term clinical studies are needed to better understand the link between oxidative stress and the clinical response to antipsychotic drugs and the therapeutic potential of antioxidants to increase the response to antipsychotics.

## 1. Introduction

Schizophrenia is recognized as a psychiatric disorder affecting numerous brain systems [1] of more than 20 million people worldwide [2]. The “neurodevelopmental hypothesis of schizophrenia” was first formulated in the 1986 and reports that abnormalities occurring during the early brain development enhance the risk to develop the clinical symptoms in adulthood [3]. Clinical symptoms observed in schizophrenic patients, such as hallucinations, delusions, negative symptoms, and cognitive deficits, often arise from neurodevelopment abnormalities. The “dopamine hypothesis” assumes that the overactivation of the mesolimbic dopamine (DA) pathway is responsible for the development of positive symptoms as a direct result of excessive DA release and activity in limbic structures, while the hypofunctioning of the mesocortical DA pathway, which projects to the frontal cortex, is proposed as the mediator of negative, affective, and cognitive symptoms of schizophrenia [4,5]. The involvement of a dopaminergic system in the pathophysiology of schizophrenia comes from different evidences, including the ability of antipsychotic drugs belonging to the class of dopamine D_2_ antagonists to reduce psychotic symptoms [6].

Recent studies suggest a primary role of oxidative stress in an early phase of schizophrenia’s pathogenesis. It has been shown that early life stress such as maternal separation or physical and/or psychological abuses increases oxidative stress phenomena at brain level, raising the risk of developing psychosis [7]. Oxidative stress refers to a phenomenon characterized by the homeostatic imbalance between the production of pro-oxidant species, such as reactive oxygen (ROS) and nitrogen (RNS) species, and the ability of antioxidant systems to neutralize them [8,9]. Oxidative stress may be involved in the early stage of the pathological course of schizophrenia [10], leading to parvalbumin-positive interneuron dysfunction in the young adult cortex [11]; indeed, the hypofunction of parvalbumin GABAergic interneurons causes an increased glutamate release, which in turn over-stimulates the activation of N-methyl-D-aspartate (NMDA) receptors and subsequent oxidative stress [4,12]. The enhanced DA activity in schizophrenia pathogenesis may be responsible for the production of ROS that, paralleled by decreased antioxidant defenses, leads to neuronal damage, contributing to the onset of the different clinical phenotypes [13]. The DA hyperactivation in different brain areas such as nucleus accumbens, caudate nucleus, and amygdala is associated with oxidative stress phenomena, in particular increased superoxide radical (O_2_^•−^) production, in schizophrenic patients [14].

It has been reported that the oxidative stress associated with first-generation antipsychotics (FGAs) treatment represents a mechanism contributing to the development of extrapyramidal side effects (EPS), especially tardive dyskinesia (TD), a potentially irreversible movement disorder [15]. The long-term treatment with FGAs (e.g., haloperidol) decreases antioxidant enzymes’ levels, then contributing to the worsening of the pro-oxidant events [16]. Moreover, haloperidol chronic treatment has been associated with the increased lipid peroxidation observed in different brain areas such as in the striatum, contributing to neuronal damage [15].

The development of second-generation antipsychotics (or atypical antipsychotics) that began 25 years ago has yielded some advances in terms of efficacy, with a relevant improvement in the treatment of negative symptoms, and in tolerability, particularly with regard to EPS [17] and only a partial improvement in the treatment of cognitive deficits [18].

According to the neuroscience-based nomenclature [19], second-generation antipsychotics include five medications with five different pharmacodynamics profiles: D_2_ receptor (D2R) antagonists (e.g., amisulpride); D_2_/5-HT_2_ receptor antagonists (e.g., olanzapine); D_2_/5-HT_1A_ receptor partial agonists (e.g., aripiprazole, brexpiprazole); D_2_/5-HT_2_/NEα_2_ receptor antagonists (e.g., clozapine); D_2_/5-HT_2_ receptor antagonist/NE reuptake inhibitors (e.g., quetiapine).

Despite the improved safety and tolerability profile compared with FGAs, the use of second-generation antipsychotic medication has been associated to unwanted side effects’ safety issues, among which metabolic alterations, such as high levels of glucose, hyperlipidemia, and obesity as well as to the development of type 2 diabetes mellitus (T2DM) and hypertension [20,21], hence affecting long-term adherence to the treatment [22,23]. In particular, clozapine or olanzapine treatment in schizophrenic children has been associated with abnormalities in cholesterol and triglycerides levels [24], while it has been associated to an increased risk of T2DM in addition to weight gain in adult schizophrenic patients [25,26].

Second-generation antipsychotic drugs may exert an antioxidant activity by reducing ROS production and lipid peroxidation in schizophrenic patients [27]. Among them, quetiapine, an antipsychotic widely used for the treatment of psychosis [28], has shown antioxidant activities preventing the alterations due to oxidative stress at the hippocampal level [29]. In addition to that, clozapine, considered the gold standard drug in treatment-refractory schizophrenia [30], is able to improve negative symptoms in patients with chronic schizophrenia through its antioxidant activity [31].

In this review, we will first examine the pro-oxidant effects of FGAs, focusing on haloperidol oxidative stress-induced neurotoxicity. We will then discuss the potential antioxidant activity of second-generation antipsychotic drugs and its relevance for future pharmacological strategies in schizophrenia.

## 2. Schizophrenia and Oxidative Stress

Schizophrenia is a complex and severe psychiatric syndrome affecting about 1% of population worldwide. Due to the lack of objective clinical tests or biomarkers, the diagnosis of this pathology is based on the observation of the subject’s clinical history and clinical phenotypes. The first episode of psychosis (FEP) generally occurs in late adolescence or during early adulthood with behavioral and cognitive alterations [32]. In particular, different clinical phenotypes can be observed including: (1) positive symptoms (hallucinations, delusions, and disorganized thoughts and speech); (2) negative symptoms (anhedonia, apathy, and social withdrawal); (3) cognitive deficits (working memory, problem solving, and executive function) [33].

Various hypothesis have been proposed to explain the pathophysiology of schizophrenia [34]; according to the “dopaminergic hypothesis”, the positive symptoms of schizophrenia result from the hyperactivation of the mesolimbic DA pathway, while a hypoactivation of the same pathway is observed in the frontal cortex cause negative and cognitive symptoms. This hypothesis is based on the observation that cocaine and amphetamines by increasing DA availability could induce psychotomimetic effects in health individuals, whereas dopamine D_2_ antagonists reduce psychotic symptoms in schizophrenic patients. This hypothesis has been recently integrated with the “glutamatergic hypothesis”, where a low activity of NMDA receptors on GABAergic interneurons might be the cause for the dopaminergic dysfunction observed in schizophrenia [35]. Indeed, the overactivation of glutamatergic system in the medial prefrontal cortex (mPFC) is considered a pathophysiological hallmark of schizophrenia and an increased glutamate efflux in prefrontal cortex (PFC) plays a significant role in the development/progression of both positive and negative symptoms [30,36,37]. Glutamate dysfunction in schizophrenia also involves alterations in intracellular molecules fundamental for glutamate receptor-associated signal transduction, including post-synaptic density protein 95 (PSD-95) [38,39,40]. PSD-95 is part of a protein family representing a pivot point for different pathways involved in the regulation of the mechanism of action of many psychotropic drugs [41]. In schizophrenic patients, a reduction in PSD-95 gene expression has been observed in PFC [42], while the PSD-95 mRNA and protein levels were increased in the occipital cortex [43] and thalamus [44], respectively. Furthermore, PSD-95-like molecules, such as synapse-associated protein 102 (SAP-102) and neurofilament light peptide (NF-L), have been proposed to be involved in the pathophysiology of schizophrenia [45], reinforcing the implication of glutamatergic dysfunction in this psychiatric disease.

Dopaminergic system is a brain’s modulatory system involved in many functions such as learning, motivation, and cognition [46], and hyperdopaminergic neurotransmission is involved in the pathophysiology of schizophrenia [47]. High levels of D2R in striatum, nucleus accumbens, and olfactory tubercle have been closely associated with positive symptoms in schizophrenia [48]. D2R blockade in post-synaptic neurons by FGA treatment plays an important role in reducing positive symptoms and acute psychosis [49] but, unfortunately, without any effect on negative and cognitive symptoms. [50]. 5-HT_2A_ antagonism in combination with D2R blockade by second-generation antipsychotic drugs results in the DA release into the PFC [33], thereby improving negative symptoms.

The dopaminergic and glutamatergic hypotheses alone are not sufficient to explain the pathophysiology of schizophrenia, partially explaining the limited efficacy observed in some cases for FGAs and second-generation antipsychotics and the research of innovative approaches alternative to the pharmacological modulation of dopamine D2 receptor activity [51].

### 2.1. The Neurobiological Link between Oxidative Stress and Inflammation in Schizophrenia

Different additional factors such as oxidative/nitrosative stress and neuroinflammation have been suggested to contribute to the etiology of schizophrenia, as well as the interaction between multiple risk genes and environmental factors [52].

Nitric oxide (NO), a gaseous free radical and one of the most representative RNS species, contributes to oxidative stress and modulates DA release, which is the reason why there is a growing interest of researchers in investigating its role in the pathophysiology of schizophrenia [53]. Clinical studies have reported the potential effect of sodium nitroprusside, a NO donor used in clinical practice to treat hypertension, in the improvement of both positive and negative symptoms in schizophrenic patients [54]. The mechanism underlying its effect might be mediated via nicotinamide adenosine dinucleotide phosphate-NO-cyclic guanosine monophosphate (NMDA-NO-cGMP) signaling activation, which in turn exerts an inhibitory effect on DA transporters, regulating the cortical DA hypofunction [4,54]. In addition, it has been shown that the co-administration of NO donors prevents haloperidol-induced TD [55], suggesting the potential of NO donors as a novel therapeutic option to prevent extrapyramidal symptoms induced by FGAs [56].

Inflammation is an essential component of the response to infection, toxic chemicals, and tissue damage. The inflammatory process taking place at the central nervous system (CNS) level (neuroinflammation), particularly in response to infection or neuronal cell injury, involves the activation of microglia with the consequent production of inflammatory mediators (pro-inflammatory cytokines and chemokines) that influence the adjacent astrocytes and neurons [57], two cell types that make an essential contribution to the homeostatic regulation of the brain tissue. Moreover, endothelial cells and perivascular macrophages are essential for the propagation of inflammatory signals within the CNS [58]. Inflammatory reactions could be both beneficial (neuroprotective) and detrimental (neurotoxic) to the CNS, depending on the interactions occurring between environmental factors, genetic variations, and various components of the inflammatory response [59]. As a consequence of the repair mechanism’s failure, an exacerbated or chronic inflammatory state underlies the progression of neurodegenerative events in different neuropsychiatric disorders including schizophrenia [60].

An excessive and prolonged microglial response leads to deleterious effects on neuronal plasticity and apoptosis, resulting in behavioral and cognitive deficits. Microglial activation along with an increased amount of these cells in the brain of schizophrenic patients have been demonstrated in post mortem studies [61]. As demonstrated by meta-analysis studies, schizophrenia is associated with immune system dysfunction, including aberrant cytokine levels [62]; in particular, it has been shown that interleukin-1β (IL-1β), interleukin-6 (IL-6), and transforming growth factor-beta 1 (TGF-β1) can be considered state markers, since they are increased during FEP, but they drop to normal levels following antipsychotic treatment; in contrast, interlukin-12 (IL-12), interferon-gamma (IFN-γ), tumor necrosis factor-alfa (TNF-α), and soluble interleukin-2 receptor (sIL-2R) may be considered as biomarkers of chronic exacerbations because their levels remain elevated even following antipsychotic treatment. In addition to the above-mentioned pro-inflammatory markers, it has been shown that the levels of an acute inflammatory phase protein, the C-reactive protein (CRP), were found increased in schizophrenic patients [63] correlating with negative symptom severity [64], underlining that the elevated blood CRP levels could be considered as a useful peripheral biomarker of schizophrenia [65].

Inflammatory responses and mitochondrial metabolic processes lead to the generation of free radicals. ROS and RNS such as hydrogen peroxide (H_2_O_2_), O_2_^•−^, hydroxyl radical (^•^OH), NO, and peroxinitrite (ONOO^•^) are highly reactive chemical species generated during normal metabolic processes and within the respiratory chain, with complex I (NADH: ubiquinone oxidoreductase) and complex III (ubiquinol: cytochrome c oxidoreductase) representing the major sources of O_2_^•−^ [34]. Oxidative and nitrosative stress processes generate alterations in the physiological redox state of cells, leading to excessive peroxides and free radical production with highly toxic effects [66]. Excessive ROS and RNS production can provoke modification of macromolecules such as nucleic acid, proteins, and lipids; in fact, these reactive species have been related to DNA mutagenesis and structure chromatin alterations (influencing gene expression), denaturation of proteins (generating non-functional proteins), lipid peroxidation (causing damage to cell membrane and cellular organelles membranes), inactivation of critical enzymes, and cell death through the activation of kinase and caspase cascades [52].

### 2.2. Oxidative Stress in Schizophrenic Patients: Role of the Antioxidant Machinery

Under physiological conditions, cellular oxidative balance is maintained by the antioxidant machinery, involving a series of enzymatic and non-enzymatic components responsible for the inhibition of ROS formation and/or free radical removal. The most representative elements part of the antioxidant enzymes are superoxide dismutase (SOD), catalase (CAT), glutathione peroxidase (GPX), and glutathione reductase (GR) [67], while albumin, uric acid, ascorbic acid (vitamin C), alfa-tocopherol (vitamin E), glutathione (GSH), and thioredoxin (TRX) belong to the non-enzymatic antioxidant molecules [68]. Both groups interact with activated oxygen and nitrogen species, counteracting the propagation of free radical chain reactions.

The above-mentioned pool of enzymes has been reported to be altered in schizophrenic patients [69]. As showed by Gawryluk et al., the levels of reduced (GSH), oxidized (GSSG), and total glutathione, the major free radical scavenger in the brain, were significantly decreased in post mortem PFC from schizophrenic patients [70]. It has been proposed by Cabungcal et al. that oxidative stress plays a central role in the development of late onset psychosis [71]. A significant reduction in the levels of GSH in peripheral tissue, cerebral fluid, PFC, and post mortem striatum of schizophrenic patients has been documented [70,72,73]; additionally, lower plasmatic levels of antioxidant molecules, such as uric acid, albumin, bilirubin, alfa-tocopherol, and ascorbic acid, have been reported [74,75,76,77]. The serum TRX levels in FEP were found to be higher when compared to chronic schizophrenic episodes following a long-term antipsychotic pharmacotherapy [78,79]. Of note, the low levels of GSH in the mPFC seems to be related to the negative symptom severity of schizophrenia [69]. A reduction in GPX and GR activity at striatum level and an increased activity of SOD in the cortex have also been detected [72,80]. Furthermore, high levels of protein oxidation have been observed in the dopaminergic areas of PFC of schizophrenic patients [81].

From all the above, it seems that oxidative stress occurs in an acute phase of schizophrenia and will persist during lifetime disease progression, then plays an important role in the pathogenesis of this disease.

## 3. First-Generation Antipsychotics (FGAs) and Oxidative Stress: The Strange Case of Haloperidol

FGAs, also known as conventional or typical antipsychotics, have been often used in schizophrenic patients for the treatment of positive symptoms, acting as DA receptor antagonists and blocking about 72% of the D2R part of the DA mesolimbic pathway [82]. It is well known that the typical antipsychotics produce EPS, including TD [55], representing the major limitation of the use of these drugs. During the FGAs pharmacological treatment, the neuroleptic malignant syndrome (NMS), a rare but fatal adverse effect [83,84], can also occur.

It has been proposed that oxidative stress may play a key role in the development of EPS and TD induced by FGAs use. TD can be due to the neuronal cell damage coming from the free radical overproduction induced by typical antipsychotics; in fact, dyskinetic patients present increased levels of lipid peroxidation products paralleled by decreased vitamin E levels [85,86,87].

Haloperidol belonging to the butyrophenones class was introduced in the clinical practice in 1960 for the treatment of acute and chronic psychosis [88], and, unfortunately, its use is associated with severe EPS, among which dystonia, parkinsonian-like syndrome, and TD are predominant [89]. Haloperidol can be converted to a free radical in the brain causing neural oxidative damage [90]. A review of the literature by Nasrallah and Chen suggests that haloperidol induces neurotoxic effects at all the considered doses, both in in vitro and in vivo studies, via different molecular mechanisms converging in neuronal cell death [91]. As showed by Gassó et al., by carrying out in vitro experiments, haloperidol significantly increased caspase-3 activity and cell death in neuroblastoma cells [92], while the chronic haloperidol administration has also shown to be able to induce neuronal apoptosis in the substantia nigra pars reticulata of rats [93]. Haloperidol administration decreases the levels of brain derived neurotrophic factor (BDNF), a neurotrophic factor implicated in neuronal survival and plasticity, reduces GSH and anti-apoptotic markers, and increases the expression of pro-apoptotic proteins in rat frontal cortex [94]. In a different study, the reduced GSH levels measured in the cortex, striatum, and cerebellum of mice were paralleled by increased NO production in the cortex [95].

The severe adverse effects due to haloperidol treatment, resulting in DA receptors blockade and neurotoxicity, have been associated to increased ROS production [96,97]. Its metabolite, haloperidol pyridiniumion, is highly toxic and increases oxidative stress inducing plasma membrane damages, explaining, at least in part, the pathogenesis of haloperidol-induced parkinsonism symptoms [98,99].

Haloperidol could also provoke oxidative stress by increasing the concentration of toxic DA metabolites, decreasing the amount of GSH, and inducting the nuclear factor kappa-light-chain-enhancer of activated B cells (NF-kB) [100]. The neurotoxicity induced by haloperidol has been related to the inhibition of survival-associated pathways such as protein kinase B (Akt) and/or the activation of caspase pro-apoptotic-mediated signals [101]. Ukai and co-workers have demonstrated that haloperidol leads to Akt inhibition and a concomitant activation of caspase-3 with subsequent neuronal death [102]. Still in the context of neurons, this antipsychotic drug can be directly toxic to neuronal cells by inducing oxidative free radicals [103,104] and indirectly by inhibiting neuronal NO-synthase (nNOS) in vitro [105].

By measuring thiobarbituric acid reactive substances, it has been demonstrated that the haloperidol chronic treatment induces oxidative damage in the brain of adult male Wistar rats [15]. In a different study, the increase in lipid peroxidation in the striatum has been observed [106]. Increased lipid peroxidation and H_2_O_2_ production paralleled by decreased activity of antioxidant enzymes (SOD, GPX, and CAT) have been found in rats with TD induced by haloperidol [107]. Another way through which haloperidol leads to oxidative stress phenomena is related to its ability to modulate cell metabolism; in fact, it has been shown that the treatment with this drug is also able to induce mitochondrial activity that in turn leads to an enhancement of ROS production in the whole blood of rats [108]. In an in vivo study carried out by Gumulec et al. using guinea pigs (*Cavia porcellus*) treated with haloperidol, two sub-groups of animals were identified according to their responses to oxidative stress; in particular, the sub-group of animals with higher haloperidol plasma levels had increased ROS production compared with the animals with lower levels of drug in their plasma [109].

Andreazza et al. demonstrated that haloperidol treatment increases lipid peroxidation in rat frontal grey matter, while it was not observed in the case of a treatment with clozapine [110]. Moreover, Kropp et al. showed the higher lipid peroxidation occurring on the plasma of schizophrenic patients treated with haloperidol when compared with second-generation antipsychotics such as clozapine and quetiapine [87]. By carrying out this 3-week longitudinal study, it was possible to underline how the oxidative stress taking place within the CNS following FGAs treatment is also reflected at peripheral level [111]

A clinical case described by Kamińska et al. regards the case of a 23-year-old woman suffering from haloperidol-induced NMS episode with increased ROS production by neutrophils and enhanced serum pro-inflammatory levels of cytokines such as IL-6 and TNF-α [112]. In an open randomized study, haloperidol treatment (5–15 mg/day) caused oxidative stress paralleled by a significant reduction in plasma antioxidant parameters (e.g., SOD) [113]. In a longitudinal, randomized, controlled, multisite, double-blind study conducted by Lieberman et al., haloperidol-treated patients with FEP exhibited a significant decrease in gray matter volume associated to neurotoxicity [114]. The treatment with FGAs may be associated with neurodegenerative phenomena in frontal areas, while the treatment with second-generation antipsychotics could be associated with neuroprotective effects, supporting their role in symptoms’ improvement [115,116]. Of note, the use of haloperidol has been associated with decreased frontal cerebral blood flow when compared to second-generation antipsychotics treatment (e.g., risperidone) [117,118].

Despite the above evidence, however, conflicting results have been reported when comparing typical versus atypical agents. A clinical trial described by Zhang et al. shows that 12 weeks of treatment with both haloperidol or risperidone reduces the elevated blood SOD levels oxidative stress-induced in schizophrenic patients [119]. In addition to that, a different study carried out by the same authors led to the conclusion that a long-term treatment with FGAs or second-generation antipsychotics (clozapine or risperidone) may induce a similar outcome on the antioxidant enzymes and lipid peroxidation levels [120]. Further long-term observational studies are needed to better understand the link between oxidative stress and the clinical response to antipsychotics drugs.

## 4. Second-Generation Antipsychotics: Can They Exert an Antioxidant Activity?

As discussed above, second-generation antipsychotics showed superior therapeutic efficacy in the management not only on positive symptoms but also on negative symptoms and cognitive dysfunctions, with few side effects [121]. The reduced risk of second-generation antipsychotics to induce EPS and TD compared to FGAs is attributed to their 5-HT_2A_ receptor antagonism and to their faster dissociation from the D2R [121]. It has been reported that second-generation antipsychotics exert neuroprotective effects by increasing BDNF levels, improving cell survival, and enhancing the neurogenesis process, also preventing or reversing the effects of haloperidol-induced toxicity [122,123].

Risperidone through its canonical antipsychotic pharmacological mechanism is able to modulate the pro-inflammatory response [124] as well as to decrease oxidative/nitrosative stress in schizophrenic patients [125]. This antipsychotic has also been shown to reduce oxidative stress and rescue synaptic plasticity in PFC pyramidal cells from a schizophrenia-like animal model obtained by perinatal phencyclidine (PCP) administration [126]. Risperidone, when administered in adolescent mice, is able to both decrease inducible nitric oxide synthase (iNOS) expression and increase CAT and SOD activity in some brain areas [127]. The antioxidant activity of risperidone is also related to its ability to increase the levels of GSH, thus improving antioxidant defense, and, at the same time, to decrease the pro-oxidant and deleterious effects of extracellular glutamate [101]. In a different study, oral risperidone administration enhanced GSH levels in the cortex and hippocampus from the PCP model of schizophrenia in which, as an additional positive outcome, the formation of lipid peroxidation products was reduced [128]. SOD enzyme is considered as the first line of defense against ROS formation and its expression may increase due to oxidative phenomena taking place in chronic schizophrenic inpatients [129], reflecting a defensive response to increased oxidative stress. Interestingly, a significant correlation has been found between the decrease in blood SOD levels and the improvement in negative symptoms in forty-one schizophrenic patients treated with risperidone [130]. These data suggest that risperidone is able to decrease in vivo the oxidative stress that characterized the brain damage observed in schizophrenic patients [16].

Along this line, a treatment for 3 months with risperidone or olanzapine significantly increased the plasma levels of the non-enzymatic antioxidants GSH, vitamin E, and vitamin C, also reducing the levels of malondialdehyde (MDA), a highly reactive compound considered a marker for oxidative stress, with an overall improvement of the antioxidant status of schizophrenic subjects [131]. In a study conducted by Hendouei et al., clozapine showed a higher antioxidant activity than risperidone or perphenazine, increasing SOD and GSH serum levels and reducing lipid peroxidation [31]. The antioxidant activity of clozapine and olanzapine is clinically relevant, since it has been reported a lower risk of free radical-induced damage, including neurological symptoms, in schizophrenic patients treated with these second-generation antipsychotics [113,132]. A chronic treatment with clozapine or olanzapine has been shown to up-regulate BDNF expression in rat hippocampus [133], and a long term treatment with clozapine has been positively associated with increased BDNF serum levels in schizophrenic patients [134], supporting the neuroprotective efficacy of these drugs [135]. A long-term treatment with these drugs is also able to regulate neuronal functions, such as neurogenesis and neuroplasticity, by the activation of extracellular signal-regulated kinases 1 and 2 (ERK1/2) and Akt pathways [136,137]. The neuroprotective activity exerted by olanzapine could also be due to its ability to up-regulate SOD enzyme, as it has been shown in vitro by using PC-12 cells [138], or to increase serum total antioxidant status with a concomitant reduction in serum MDA levels in schizophrenic patients [139]. Among second-generation antipsychotic drugs, clozapine and olanzapine were also reported to act as good scavengers of O_2_^•−^, one of more representative ROS [140,141]. The direct antioxidant activity of these antipsychotics contributes to their therapeutic activity [142], and it has been suggested how their ability to decrease ROS production by neutrophils may depend on the amino group part of their chemical structure [140]. The treatment with clozapine, quetiapine, or risperidone for 21 days decreases the plasma levels of MDA compared to that observed in haloperidol-treated patients [87].

When considering second-generation antipsychotics, aripiprazole acts as a partial agonist on D_2_, D_3_, and 5-HT_1A_ receptors, in addition to being a 5-HT_2A_ antagonist, leading to the improvement of positive and negative symptoms as well as of the cognitive impairment observed in schizophrenia [143,144,145]. In a recently published study, aripiprazole did not induce significant changes in plasma lipid peroxidation at the pharmacological doses commonly used for the management of acute episode, while, unexpectedly, it showed antioxidant effects when used at low doses [144]. A different study showed that aripiprazole has positive effects on depression-induced oxidative stress in rat brain [146]. Interestingly, a case report of a 51-year-old schizophrenic Korean woman described by Hue and Lee demonstrated that low-dose aripiprazole is effective in the management of clozapine-associated TD [147]. In addition, the co-administration of lithium and aripiprazole has been shown to be effective in decreasing oxidative stress in subjects with bipolar disorder [148]. Furthermore, this drug decreased lipid peroxidation and increased SOD2 enzyme levels in the dentate gyrus of adolescent mice [149].

Overall, these data suggest that second-generation antipsychotics such as risperidone, clozapine, and olanzapine possess a clinically relevant ability to improve the antioxidant machinery, which might play a significant role in the treatment of schizophrenia.

Nowadays, the COVID-19 pandemic represents a massive world public health problem caused by severe acute respiratory syndrome coronavirus 2 (SARS-CoV-2) [150]. This disease is characterized by oxidative stress [151] and neuroinflammation [152] along with a higher risk to develop delirium and psychomotor agitation [153]. Among the second-generation antipsychotics, quetiapine, risperidone, and especially injectable aripiprazole have been proposed as alternative treatment choices for COVID-19 patients with delirium [153,154]. Since these second-generation antipsychotics have shown antioxidant and anti-inflammatory activities, it would be interesting to assess, in future studies, whether schizophrenic patients treated with these drugs might be less susceptible to detrimental COVID-19-related effects.

### Antioxidant Treatments in Schizophrenia

As mentioned above, an increasing number of studies have pointed toward an association between the occurrence of oxidative stress and the risk to develop psychosis. There are numerous preclinical studies highlighting the therapeutic potential of antioxidants for the treatment of FEP [155], a critical intervention period in schizophrenia. One of the most used antioxidants is represented by *N*-acetylcysteine (NAC) that has been shown to counteract schizophrenia-like bio-behavioral changes in rats exposed to maternal immune [156], prevent the loss of cortical inhibitory parvalbumin-positive interneurons [71], and reverse dysregulated mitochondrial activity and the related production of ROS/RNS [157] in different animal models of schizophrenia. Other antioxidants that have been considered in preclinical studies, in particular to manage psychotic symptoms, are represented by apocynin, omega-3 fatty acids, vitamin C, and ebselen [155].

Moving from preclinical to clinical studies, a review by Magalhães et al. evaluated the therapeutic potential of antioxidants as add-on treatments to standard antipsychotic medication in subjects suffering from schizophrenia [158]. In this study, considering 22 randomized controlled trials involving people with schizophrenia in whom the effects of seven different antioxidants were analyzed, *Ginkgo biloba* and NAC emerged as the most promising add-on treatments even though the authors concluded that there is a need for larger trials with longer periods of follow-up to be conducted. The benefits coming from the use of NAC as adjunctive antioxidant supplementation have also been observed in FEP [159]; in particular, in FEP the administration of NAC improved the oxidative status that was paralleled by reduced psychotic symptoms.

## 5. Effects of Second-Generation Antipsychotics on Microglia: Therapeutic Potential for the Treatment of Schizophrenia

**Figure 1:**
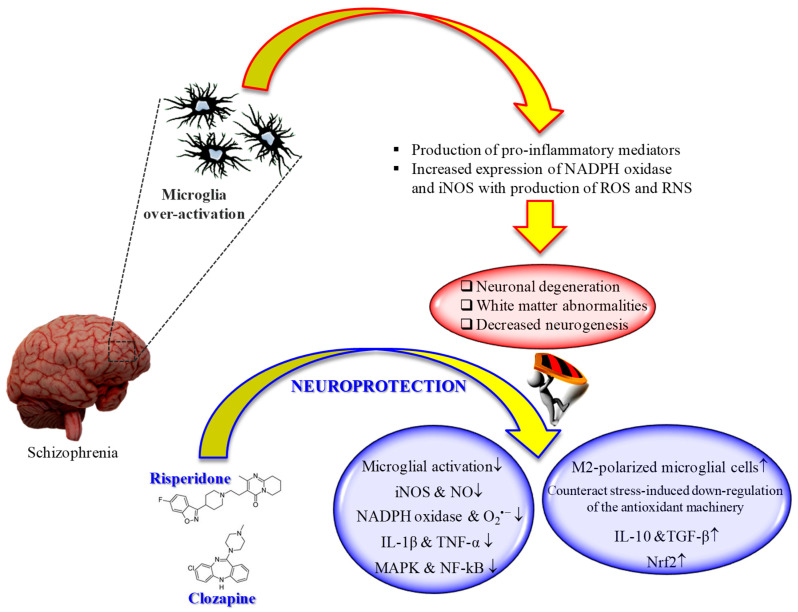
“Microglia hypothesis of schizophrenia”: therapeutic potential of second-generation antipsychotics. ↑ = increased; ↓ = decreased.

It is well known that microglia, the tissue-resident macrophages of the brain and spinal cord [160], represent an important contributor of both inflammation and oxidative stress [161,162]. In fact, microglial cells, as a response to the pathological changes occurring at brain level, produce pro-inflammatory mediators as well as ROS and RNS, leading to the neuronal degeneration, white matter abnormalities, and decreased neurogenesis observed in schizophrenia [163]. That led to the development of “microglia hypothesis of schizophrenia” [163,164] (Figure 1).

Several studies have shown the therapeutic potential of second-generation antipsychotics on the modulation of microglia activity, with specific regard to pro-inflammatory cytokines and reactive species release.

An in vivo study carried out by MacDowell et al. showed that paliperidone (1 mg/kg i.p.) has the potential to regulate the antioxidant and anti-inflammatory pathways in a rat model of acute and chronic restraint stress [165]. In particular, the drug was able to up-regulate nuclear factor erythroid-related factor 2 (Nrf2) and antioxidant response element-dependent antioxidant enzymes during acute stress conditions, while in chronic stress counteracted stress-induced down-regulation of the endogenous antioxidant machinery. Of note, paliperidone was also able to increase TGF-β and interleukin-10 levels as well as the number of M2-polarized (anti-inflammatory phenotype) microglial cells in acute and chronic stress conditions.

In an in vitro model of neuroinflammation represented by phorbol 12-myristate 13-acetate (PMA)-treated microglia, aripiprazole inhibited O_2_^•−^ formation by the activation of protein kinase C (PKC) and intracellular Ca^2+^ regulation, reducing oxidative reactions [166]. In the same study, the authors showed the ability of aripiprazole to reduce the formation of neuritic beading, then to protect neurons against the damage induced by PMA-stimulated microglial activation. The same research group was also able to demonstrate that aripiprazole significantly inhibited the release of NO and pro-inflammatory cytokines from microglial cells treated with IFN-γ [167]. The results of these works are relevant, since the overproduction of both NO and O_2_^•−^ has been associated with the pathogenesis of schizophrenia [168].

Different in vivo and in vitro studies have highlighted the potential beneficial role of risperidone in the case of microglia-induced oxidative stress (Figure 1). This second-generation antipsychotic may exert its antioxidant and anti-inflammatory activity via the inhibition of NO and pro-inflammatory cytokines production by activated microglia [169]. In 2013, MacDowell and colleagues, by using an in vivo model of mild neuroinflammation represented by a lipopolysaccharide (LPS)-treated (0.5 mg/kg i.p.) young adult rat, demonstrated the double effect of risperidone in counteracting both oxidative stress and inflammation; from one hand this drug was able to prevent the increased expression LPS-induced of interleukin IL-1β and TNF-α, and the activity of iNOS and cyclooxygenase, p38 mitogen-activated protein kinase (MAPK) and NF-kB in brain cortex, one the other hand it restored anti-inflammatory pathways consisting in deoxyprostaglandins and peroxisome proliferator activated receptor γ decreased by LPS challenge [170]. Still, in the context of risperidone neuroprotective potential, Zhu et al. investigated the ability of this drug, alone or in combination with minocycline, to prevent microglia activation and counteract schizophrenia-like behavioral deficits in intrahippocampal LPS-injected rats [171]. The authors were able to show that both drugs, alone or in combination, attenuated the behavioral alterations and inhibited the dramatic microglia activation LPS-induced in ventral hippocampus, ventrobasal thalamus, and cerebral cortex.

During the last decade, the ability of clozapine to modulate microglial activation and the related oxidative stress phenomena has been well investigated (Figure 1). It is well known that excessive ROS produced by NADPH oxidase in over-activated microglia can lead to neuronal death [172]. Shin et al. provided a possible mechanism by which clozapine may interfere with ROS production and the consequent oxidative stress, corresponding to the inhibition of proton currents in microglia, required to maintain NADPH oxidase activity [173]. Of note, of the three drugs used by the authors in their study, only clozapine was able to reach the concentration needed to inhibit microglial proton currents in the brain at therapeutic doses. The antioxidant activity of clozapine was also observed in a social isolation rearing (SIR) rat model, in which the behavioral disturbances and cortico-striatal SIR-induced oxidative stress were corrected by clozapine administration [174]. As showed by Hu et al. by using primary cortical and mesencephalic neuron-glia cultures, clozapine is able to protect dopaminergic neurons from LPS-induced damage by inhibiting microglial overactivation [175]. In particular, clozapine decreased neurotoxicity and microglial activation, as well as the production of microglia-derived NO, O_2_^•−^, and total intracellular ROS; additionally, clozapine pre-treatment inhibited the translocation of cytosolic subunit p47-phox to the membrane in microglia induced by LPS. Not just clozapine but also its metabolites have shown to protect neurons against the microglia-induced oxidative stress. In fact, as shown by Jiang and co-workers, clozapine N-oxide (CNO) and *N*-desmethylclozapine (NDC), two clozapine metabolites, are able to exert neuroprotection through the inhibition of microglial NADPH oxidase [176]. The authors were able to link the observed neuroprotection following CNO and NDC treatment to the inhibition of NADPH oxidase and microglia-mediated release of pro-inflammatory mediators. In a different study, Ribeiro et al. attempted to determine the progressive inflammatory and oxidative alterations induced in rats by the administration of polyriboinosinic–polyribocytidilic acid (poly(I:C)) and its possible reversal by the administration of clozapine [177]. Interestingly, the progressive microglial activation and iNOS increase accompanied by deficits in prepulse inhibition of the startle and polyI:C-induced working memory were all reversed by the administration of clozapine.

## 6. Conclusions and Perspectives

Nowadays, many people worldwide suffer from schizophrenia, a psychiatric disorder characterized by recurrent psychotic episodes with oxidative stress exerting a key role in the pathogenic process that contributes to declining course and poor outcome in this disease. Among the cells populating the brain, microglia significantly contribute to oxidative stress and the inflammatory phenomena observed in an early phase of schizophrenia patients, which is the reason why “microglia hypothesis of schizophrenia” has been recently proposed. Several in vitro and in vivo studies have shown the therapeutic potential of second-generation antipsychotics, such as risperidone and clozapine, on the modulation of microglia activation and oxidative stress. In particular, these drugs, presenting improved safety and tolerability profile compared to FGAs, have been able to decrease microglial activation and the related oxidative stress, also enhancing the endogenous antioxidant machinery. Since microglia-induced oxidative stress has been associated with neuronal degeneration, white matter abnormalities, and decreased neurogenesis observed in schizophrenia, the evidence discussed in the present review suggests that the increased therapeutic efficacy of second-generation antipsychotics compared to FGAs might be linked to their clinically relevant antioxidant activity. Further long-term clinical studies are needed to better understand the link between oxidative stress and the clinical response to antipsychotics drugs and the therapeutic potential of antioxidants to increase the response to second-generation antipsychotic drugs. Given that increasing the treatment adherence with current available second-generation antipsychotics remains an unmet clinical need, future observational studies will help to clarify the relevance of the antioxidant activity to improve the tolerability and safety profile of these drugs in clinical practice.
